# Case report: Primary cardiac lymphoma manifesting as superior vena cava syndrome

**DOI:** 10.3389/fcvm.2023.1257734

**Published:** 2023-09-22

**Authors:** Joseph Kassab, Georges Gebrael, Michel Chedid El Helou, Joseph El Dahdah, Elio Haroun, Rebecca Kassab, Saad Abou Ali, Ziad Khabbaz, Roland Kassab

**Affiliations:** ^1^ Cleveland Clinic Foundation, Heart Vascular and Thoracic Institute, Cleveland, OH, United States; ^2^ Department of Medicine, Saint Joseph University of Beirut, Achrafieh, Beirut, Lebanon; ^3^ Department of Cardiovascular Medicine, Sacre Coeur Hospital, Baabda, Beirut, Lebanon; ^4^ Department of Cardiovascular Medicine, Hotel-Dieu De France, Achrafieh, Beirut, Lebanon

**Keywords:** lymphoma, cardiac lymphoma, primary cardiac lymphoma, superior vena cava syndrome, diffuse large B cell lymphoma

## Abstract

A 64-year-old man presented with symptoms indicative of superior vena cava syndrome. Imaging work-up revealed an obstructing right atrial mass, which was subsequently excised and diagnosed as primary cardiac lymphoma. Post-surgery, the patient showed significant clinical improvement and was started on a chemotherapy regimen with complete remission at 1 year.

## History of presentation

A previously healthy 64-year-old man, presented to the emergency department with 2 weeks of increasing exertional dyspnea, worsening orthopnea, facial and arm swelling, and a nonproductive cough. Vital signs on admission were within normal limits. On physical examination, the patient demonstrated marked facial and upper extremity edema, as well as distended neck veins. Additionally, signs of plethora and cyanosis of the face were noted, with an increase in jugular venous pressure on cardiovascular examination.

## Past medical history

The patient had no significant past medical history.

## Differential diagnosis

Given the patient's symptoms suggestive of superior vena cava syndrome (SVC), a broad differential diagnosis were considered. Malignant causes included primary or secondary cardiac tumors, lung cancer, mediastinal tumors, or lymphoma, which may obstruct or compress the superior vena cava. Non-malignant etiologies encompassed SVC thrombosis, aortic aneurysm, or fibrosing mediastinitis. Cardiopulmonary conditions such as heart failure, pericardial disease, and pulmonary hypertension were also suggested, given the symptoms of dyspnea and orthopnea.

## Investigations

**Figure 1:**
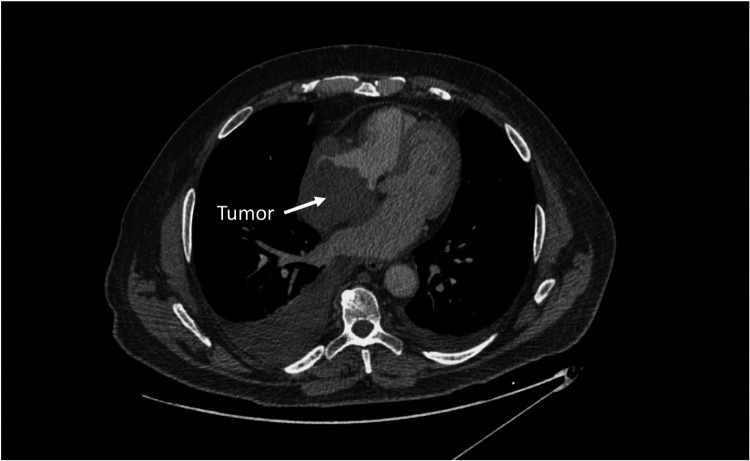
Chest CT scan demonstrating a large right atrial mass.

**Figure 2:**
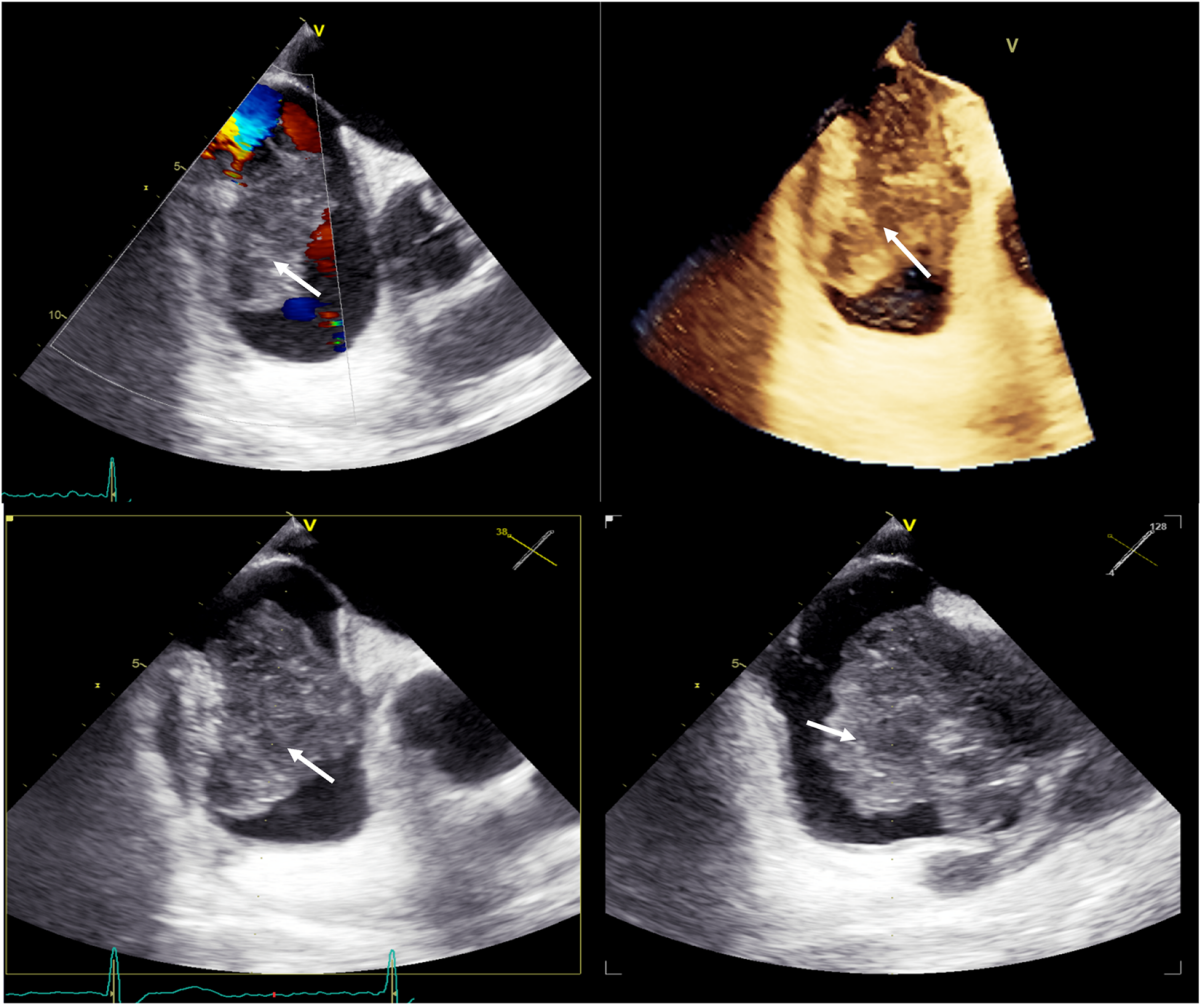
Baseline transesophageal echocardiography with 3D reconstruction, showing a large tumor arising from the lateral wall of the right atrium (white arrows).

**Figure 3:**
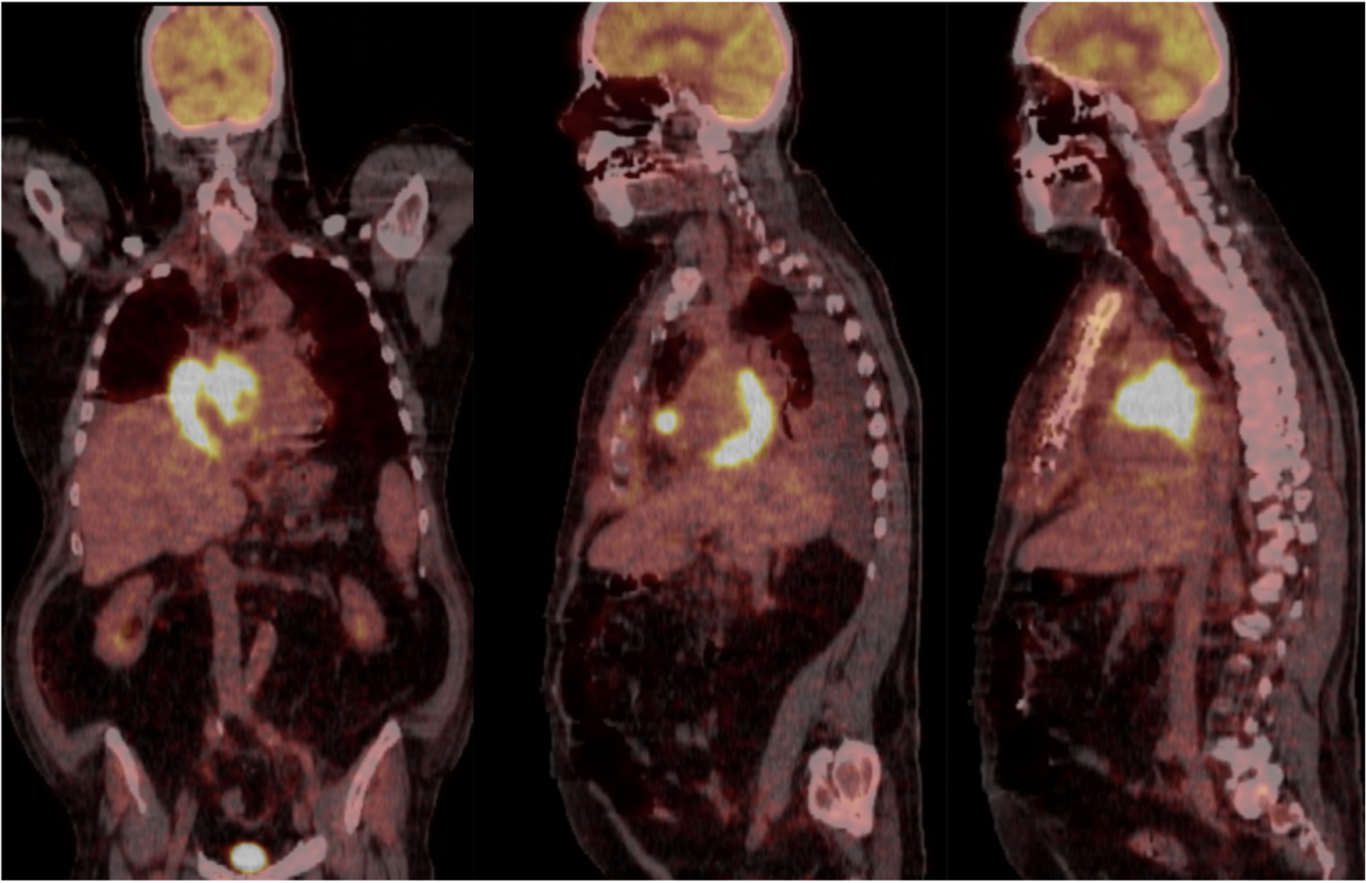
Whole-body positron emission tomography/computed tomography (PET/CT) in coronal and sagittal views showing pronounced uptake of fluorine-18 fluorodeoxyglucose in the right atrium, with no other metabolically active lesions.

The patient underwent a chest CT-scan, followed by a confirmatory transesophageal echocardiogram (TEE) which revealed the presence of a prominent, heterogeneous, partially non-enhancing, right atrial mass, measuring 66 × 41 × 37 mm, partially disrupting inferior vena cava flow and obstructing the superior vena cava (Figures 1, 2, Supplementary Video S1). A filling defect was also noted in the proximal right pulmonary artery, suggesting emboli origination from the tumor. A whole-body positron emission tomography/computed tomography (PET/CT) showed pronounced uptake of fluorine-18 fluorodeoxyglucose in the right atrium, with no other metabolically active lesions (Figure 3, Supplementary Figure S1).

## Management

**Figure 4:**
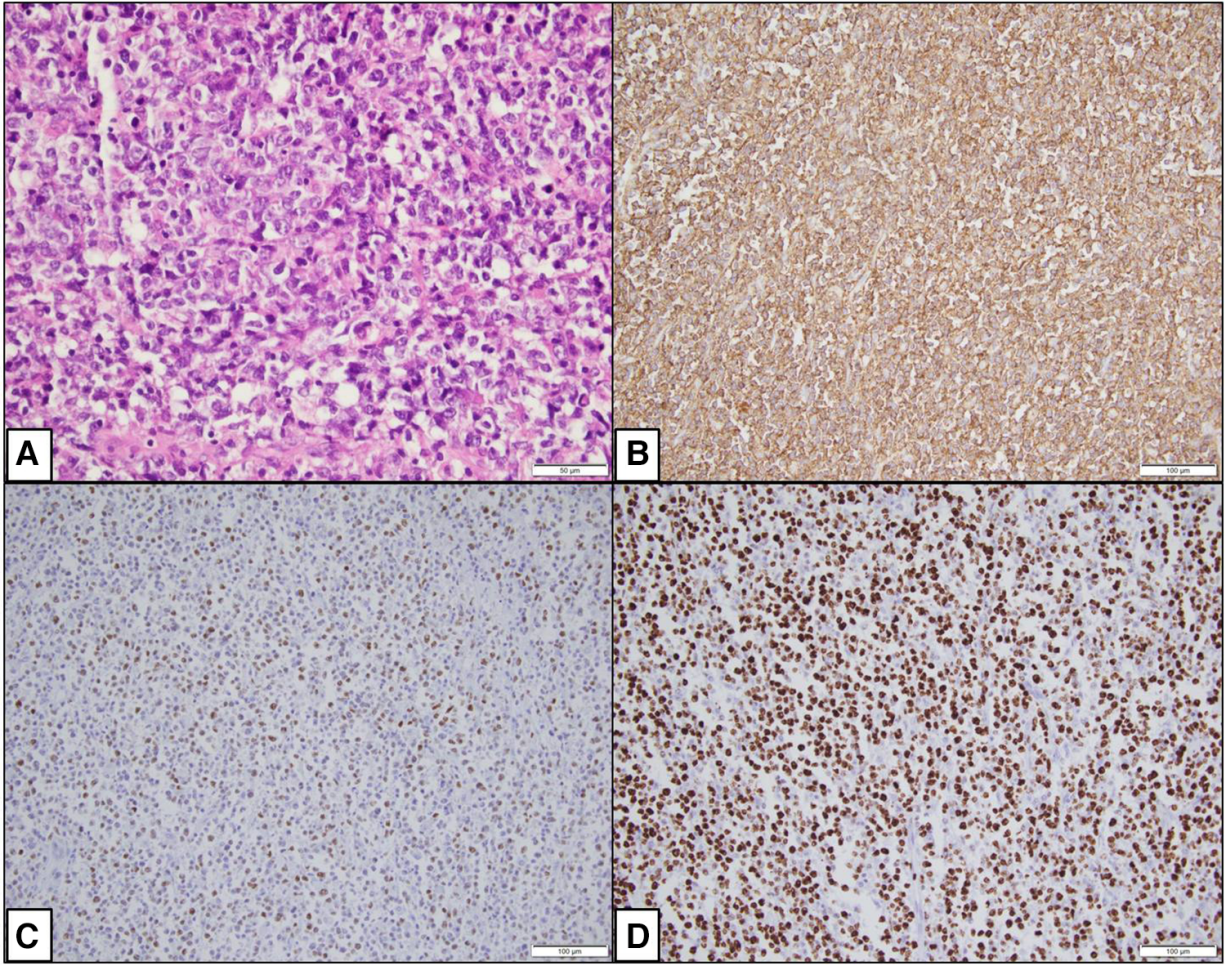
Photomicrographs showing histopathological changes. (**A**) (H&E × 400): Microscopy shows a diffuse proliferation of large atypical cells. (**B**) (IHC × 200): CD20 is positive in the large lymphocytes. (**C**) (IHC × 200): MUM1 is positive in the large lymphocytes. (**D**) (IHC × 200): Ki67 is approximately 95%.

Given the high risk of deteriorating clinical condition, surgical removal of the mass was planned and successfully executed. The surgical specimen (Supplementary Figure S2) was then sent to the pathology lab for microscopic examination. This examination showed a diffuse proliferation of large atypical lymphocytes exhibiting mitotic activity and apoptosis along with extensive areas of necrosis. Immunohistochemistry staining tested positive for CD20 (Figure 4), leading to a diagnosis of right atrial diffuse large B cell lymphoma, non-germinal center (activated) type. This confirmed the diagnosis of primary cardiac lymphoma (PCL). Following surgery, the patient's clinical condition improved significantly, and he was subsequently started on a R-CHOP chemotherapy regimen (intravenous rituximab, cyclophosphamide, doxorubicin, vincristine, and prednisolone).

## Discussion

While secondary cardiac lymphomas are relatively frequent and can contribute to 20% of extra nodal non Hodgkin lymphomas (NHL), primary cardiac lymphoma (PCL) is extremely rare and represents only 1%–2% of all heart tumors (1), since the cardiac tissue contains little to no lymphocytes. Almost all of PCLs appear to be derived from B-cell lineage with diffuse large B cell lymphoma (DLBCL) being the most common type, whereas other types such as Burkitt lymphoma or chronic lymphocytic leukemia/small lymphocytic lymphoma are less likely to be reported (1). Although immunosuppression (e.g., transplant recipients, HIV patients, immunosuppressant drugs) could be a risk factor for developing PCL, it usually occurs in immunocompetent adults with a median age of presentation of 55–65 years and 65%–85% of all cases in males (2)—features compatible with our patient. PCL may present with a wide range of symptoms, generally depending on the site of involvement of the heart, size and growth rate. These symptoms may encompass signs of heart failure, such as shortness of breath and pedal edema, as well as chest pain, arrhythmia (particularly atrial fibrillation and AV block), constitutional symptoms (e.g., fever, chills, night sweats, weight loss), and SVC syndrome as was seen in our patient (2, 3). All heart chambers as well as the interatrial and the interventricular septum may be involved; however, the tumor tends to arise in the wall of the right heart, specifically in the right atrium, with epicardial and pericardial involvement in about 50% of cases (4).

Differential diagnoses include benign myxoma (the most common type of cardiac tumor), intracardiac thrombi (in the setting of hypokinetic dilated cardiomyopathy) and angiosarcoma, (the most common malignant heart tumor, usually found in the left cavities) (5). Initial evaluation of cardiac masses often involves noninvasive multimodality imaging, including 2D or 3D echocardiography, MRI, and contrast CT scan, depending on diagnostic suspicion (6). As was done with our patient, transesophageal echocardiography is a key initial diagnostic tool for differentiating diagnosis and guiding further imaging and management. Cardiac CT scan and MRI are used to determine the extent of heart involvement and guide biopsy and/or surgical approach. PET scan may also be performed to assess for primary tumors or extracardiac involvement—which was negative in this case confirming the diagnosis of PCL. Histopathological confirmation is required for definitive diagnosis, and cytological examination is sometimes helpful in the presence of pericardial effusion.

The mainstay of PCL treatment relies on chemotherapy, using the combination of Rituximab with cyclophosphamide, doxorubicin, vincristine, and prednisolone (R-CHOP regimen), which has shown a response rate of 79%–87% (5). Prompt initiation of chemotherapy often provides symptomatic relief as well as complete remission. Side effects include tumor lysis syndrome and sepsis in around 10% of cases. In addition, chemotherapy can lead to massive thromboembolism, cardiac wall perforation, ventricular septal rupture, life threatening arrhythmias and pericardial effusion. For these reasons, some reports suggest that reduced doses of chemotherapy “R-mini-CHOP” in the initial course of treatment could reduce the risk of sudden cardiac death (7). Radiation therapy may be used in combination with chemotherapy, particularly in refractory cases, and reports have shown improved survival. However, it has some limitations given the risk of radiation-induced heart disease including pericarditis, cardiomyopathy, coronary artery disease, conduction defects and diastolic dysfunction (8). While surgical management is not the mainstay of treatment, prompt surgical debulking is indicated in patients with acute and severe presentations, including SVC syndrome (as in our patient) (9) and rapidly progressive heart failure (10). The overall prognosis of PCL is generally favorable, with a remission rate of 61% following treatment with chemotherapy alone (10).

## Follow up

The patient is currently in remission at 1-year follow-up.

## Conclusion

PCL is an extremely rare neoplasm. While chemotherapy remains the mainstay of treatment, surgery could be considered for urgent symptomatic relief.

## Data Availability

The original contributions presented in the study are included in the article/**Supplementary Material**, further inquiries can be directed to the corresponding author.

## Supplementary material

Supplementary Figure 1 Whole-body positron emission tomography/computed tomography (PET/CT) in axial view showing pronounced uptake of fluorine-18 fluorodeoxyglucose in the right atrium, with no other metabolically active lesions.

Supplementary Figure 2 Surgical specimen of the right atrial mass (white arrow).

Supplementary Video 1 Baseline transesophageal echocardiography, showing a large tumor arising from the lateral wall of the right atrium invading and causing significant obstruction of the superior vena cava.
