# Supplementary figures and images for: Case report: Primary cardiac lymphoma manifesting as superior vena cava syndrome
Source: Front Cardiovasc Med. 2023 Sep 22;10:1257734. doi: 10.3389/fcvm.2023.1257734 (PMC10556236; doi:10.3389/fcvm.2023.1257734)

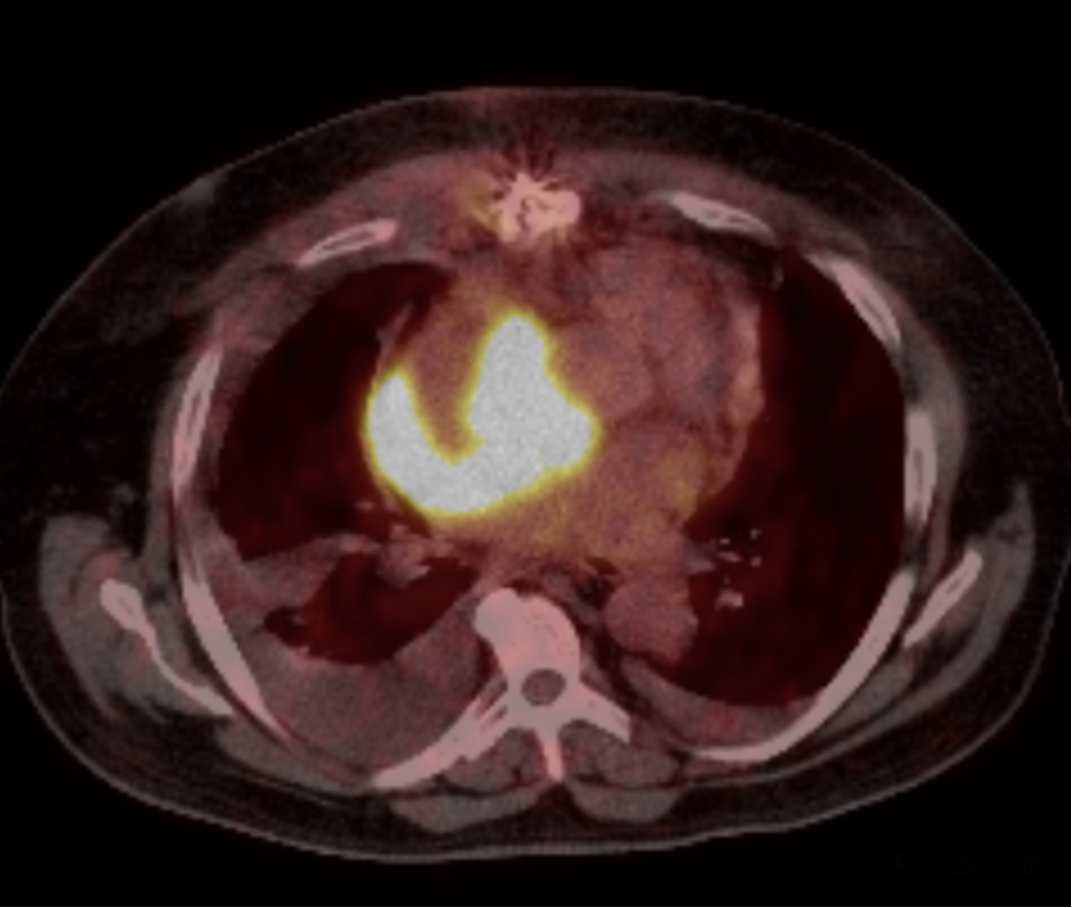

Supplement: Supplementary Figure 1 — Whole-body positron emission tomography/computed tomography (PET/CT) in axial view showing pronounced uptake of fluorine-18 fluorodeoxyglucose in the right atrium, with no other metabolically active lesions. [file Image1.jpeg]

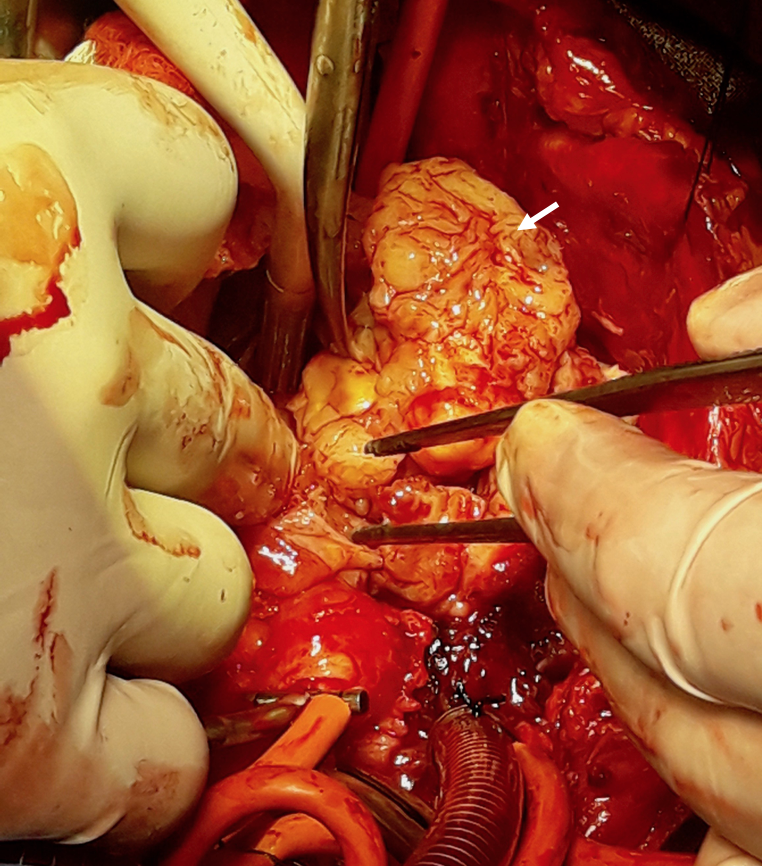

Supplement: Supplementary Figure 2 — Surgical specimen of the right atrial mass (white arrow). [file Image2.jpeg]
